# Supplementary material for: Malaria preventive practices and delivery outcomes: A cross-sectional study of parturient women in a tertiary hospital in Eastern Uganda
Source: PLoS One. 2020 Aug 7;15(8):e0237407. doi: 10.1371/journal.pone.0237407 (PMC7413511; doi:10.1371/journal.pone.0237407)
Supplement: Attachment — Submitted filename: Response to the reviewer 02.docx [file pone.0237407.s002.docx]

**Response to the reviewer’s comments**

| **SN** | **COMMENT** | **RESPONSE** |
| --- | --- | --- |
| 1 | Dear Mr Iramiot,  Thank you for submitting your manuscript to PLOS ONE. After careful consideration, we have decided that your manuscript does not meet our criteria for publication and must therefore be rejected.  Specifically, the references  do not meet the journal requirements. Kindly ensure the referencing is consistent to PLOS One guidelines. | This has been amended. All referencing is now consistent to PLOS One guidelines |
| 2 | The manuscript can be resubmitted after the corrections I am sorry that we cannot be more positive on this occasion, but hope that you appreciate the reasons for this decision. | Thank you very much |
| 3 | Additional Editor Comments (if provided):  The authors are should review their references and ensure it is consistent with journal requirement. Apart from Reference #1 and 10, all the others have to be revised. | This has been amended. All referencing has been revised and consistent to PLOS One guidelines |
| 4 | The opening statement on malaria mortality has to be revised. This is needs to be corrected before resubmission | Corrections have been made as recommended, it now reads, ‘‘Africa carries a disproportionately high burden of malaria globally,’’ thank you. |
|  |  |  |
