# Supplementary material for: Malaria preventive practices and delivery outcomes: A cross-sectional study of parturient women in a tertiary hospital in Eastern Uganda
Source: PLoS One. 2020 Aug 7;15(8):e0237407. doi: 10.1371/journal.pone.0237407 (PMC7413511; doi:10.1371/journal.pone.0237407)
Supplement: Attachment — Submitted filename: PONE-D-20-13366.docx [file pone.0237407.s003.docx]

15^th^ June/2020

To: Editorial Manager,

PLOS ONE

**Subject: A review of a manuscript “Malaria Preventive Practices and Delivery Outcomes: A Cross-sectional Study of Parturient Women in a Tertiary Hospital in Eastern Uganda.”**

Dear,

Here are the comments

Major Comment

1. Almost all references in background part are wrong. The references mentioned are not providing the stated concept. Some are directly copied from the background of other studies, with no change in reference.

Dear authors, please make sure that you are using a reference because of the finding/conclusion of that work, not because of the background they used.

Minor comments

1. The background in abstract is **not sufficient**, it says nothing about the case in pregnant women, except what is mentioned as objective
2. If possible, **avoid abbrevations** in abstract or use in the expanded form in their first appearances eg. ANC
3. It is better to use country or city as keyword for easy searchability of the work. Eg. Uganda
4. Exclusion: it doesn’t give sense to exclude deliveries on weekends and public holidays in a research(specially funded one). **Rationalize** as this exclusion may rise the idea of bias. What was your reason?
5. Grammar and spelling errors: it needs **language editing**.

Examples: **His** could be….. instead of **This** could be….. **5^th^ line of discussion**

Higher **that** others…… instead of Higher **than** others….**last line of conclusion**

6. Conclusion: in the statement saying *the effective use of malaria preventive strategies (IPT-SP and ITN) is* ***generally still low****, please do not use generally and still together. Use iether of them. I recommend* ***is generally low.***

***Recommendation:*** *Consider for the publication after correction. I will not recommend for the publication at this stage due to the references.*
