# Supplementary material for: Malaria preventive practices and delivery outcomes: A cross-sectional study of parturient women in a tertiary hospital in Eastern Uganda
Source: PLoS One. 2020 Aug 7;15(8):e0237407. doi: 10.1371/journal.pone.0237407 (PMC7413511; doi:10.1371/journal.pone.0237407)
Supplement: Attachment — Submitted filename: Response to Reviewers.docx [file pone.0237407.s004.docx]

**Response to reviewers**

| **SN** | **Comment** | **Response** |
| --- | --- | --- |
| **Reviewer 1** | | |
| **Major comments** | | |
| 1 | Almost all references in background part are wrong. The references mentioned are not providing the stated concept. Some are directly copied from the background of other studies, with no change in reference.  Dear authors, please make sure that you are using a reference because of the finding/conclusion of that work, not because of the background they used | All references have been reviewed and well aligned as recommended by the reviewer. Thank you |
| **Minor comments** | | |
| 1 | The background in abstract is not sufficient, it says nothing about the case in pregnant women, except what is mentioned as objective | Corrections have been made as recommended by the reviewer. |
| 2 | If possible, avoid abbreviations in abstract or use in the expanded form in their first appearances eg. ANC | Amended as recommended by the reviewer |
| 3 | It is better to use country or city as keyword for easy searchability of the work. Eg. Uganda | Thank you for the guidance. This has been amended. |
| 4 | **Exclusion:** it doesn’t give sense to exclude deliveries on weekends and public holidays in a research (specially funded one). Rationalize as this exclusion may rise the idea of bias. What was your reason? | This has been corrected. The recruitment was actually done throughout the week. |
| 5 | Grammar and spelling errors: it needs **language editing**.  Examples: **His** could be….. instead of **This** could be….. **5^th^ line of discussion**  Higher **that** others…… instead of Higher **than** others….**last line of conclusion** | Critical language editing has been done throughout the manuscript. Thank you. |
| 6 | Conclusion: in the statement saying *the effective use of malaria preventive strategies (IPT-SP and ITN) is* ***generally still low****, please do not use generally and still together. Use either of them. I recommend* ***is generally low.*** | This has been corrected as recommended by the reviewer. |
| **Reviewer 2** | | |
| **1** | The study is interesting and noteworthy, and contributes to the needed body of evidence about access to and impact of malaria in pregnancy interventions in sub-Saharan Africa. That said, the rigor of the IPTp coverage data is ambiguous. They refer to women who did vs did not receive X doses of IPTp with SP, while simultaneously noting that over 85% received SP by prescription, not by DOT. The researchers do not meaningfully address the rigor of self-reported IPTp administration. | Thank you. Amendments have been made for clarity. Self-reported IPTp administration has been acknowledged as a limitation to the study. |
| **2** | The data is analyzed comparing women receiving vs not receiving IPTp, however there should be some acknowledgement that there may be differences between women who received IPTp by DOT, and that there may be differences in outcome between women who received IPT via DOT vs not (noting that if IPTp is not administered via DOT, it cannot be confirmed as taken). I recognize that it would be difficult to disaggregate the IPTp data for analysis by DOT vs non-DOT, as the DOT group is likely too small to produce significant results, however the data as presented may mislead readers about the relationship between IPTp and anemia, parasitemia and birth outcome. | The authors have acknowledged this as a limitation to the study |
| **3** | There are several other minor revisions that should be made, such as updating the WHO recommended number of ANC visits to be in line with current guidelines. I propose that the recommendation “Sensitizing women about IPT during health talks will encourage women to demand for DOT mode of IPT.” should be revised to focus on changing provider behavior. Providers are responsible for the care they offer and it is not reasonable to assume that pregnant women are empowered enough and have adequate agency to demand this service. | Amendments have been made as recommended by the reviewer. |
| **4** | Additionally, the differences in detection rates of parasitemia with smears vs RDT should be considered for a potentially recommendation. | This has been improved in the discussion section and also a has been drawn from our findings and from previous studies. |
| **5** | The paragraph beginning “Of the 19 mothers with positive malaria Rapid Diagnostic Test (MRDT) at delivery” could be revised for clarity between the groups. | This has been revised as advised by the reviewer. |
| **6** | Several other places could benefit from revisions for clarity including “The women who took 2 or more doses had a higher likelihood of being compared to those who had less than two doses” and “Studies done by The difference in the prevalence between microscopy and RDT…” | The authors have recommended a longitudinal study to related IPTp uptake to the delivery outcome |
