# Supplementary material for: Malaria preventive practices and delivery outcomes: A cross-sectional study of parturient women in a tertiary hospital in Eastern Uganda
Source: PLoS One. 2020 Aug 7;15(8):e0237407. doi: 10.1371/journal.pone.0237407 (PMC7413511; doi:10.1371/journal.pone.0237407)
Supplement: Attachment — Submitted filename: Response to Reviewers.docx [file pone.0237407.s005.docx]

Response to Reviewers

| **SN** | **Comment** | **Response** |
| --- | --- | --- |
| 1 | Please remove any funding-related text from the manuscript and let us know how you would like to update your Funding Statement. | Amendments have been made as advised by the reviewer |
|  |  |  |
